# O16 Learning from acute COVID-19in a patient with potential immune dysregulation

**DOI:** 10.1093/rap/rkaa054.004

**Published:** 2020-11-03

**Authors:** Lucy Paterson-Brown, James Brighouse, Nick Wilkinson

**Affiliations:** r1 Evelina London Children's Hospital, London, United Kingdom; r2 St Helier Hospital, London, United Kingdom

## Abstract

**Case report - Introduction:**

Throughout the COVID-19 pandemic, children and adolescents have been at minimal risk of admission to hospital and intensive care from SARS-COV2 and exhibited a different phenotype of critical illness to adults. We present a 9-year-old Caucasian boy who, early in the pandemic, became PCR positive and was admitted to paediatric intensive care (PICU) with an adult phenotype of hyperinflammation and acute respiratory distress syndrome (ARDS). He has trisomy 21 and last year was admitted to PICU with ARDS and macrophage activation syndrome. We share how this early case influenced our thinking and subsequent management of cases thought to have PIMS-TS.

**Case report - Case description:**

He presented to a district hospital on March 31st with fever and persistent cough without respiratory or other compromise. Discharged home with oral antibiotics, he obtunded within 18 h and returned as an emergency with persistent fever, cough, and haemoptysis with Sa0_2_ 77% in 15litres oxygen. Following intubation, a large pulmonary haemorrhage made ventilation difficult and three cardiac arrests occurred prior to transfer on inotropic support.

On arrival, results showed Lymphocytes 1.2, Hb 104, Platelets 365, Ferritin 2059, INR 1.2, Procalcitonin (PCT) 43.84, CRP 219, CK 296, LDH 2035, Albumin 35, D-dimer 37.55. SARS-COV2 was confirmed in endotracheal secretions. All other microbiology investigations remained negative other than a scanty growth of Candida. Radiology demonstrated bilateral opacifications and pleural effusions. Blood film was consistent with the clinical picture with no signs of malignancy. Subsequent key results included cytopenia with troughs of Hb 86, Lymphocytes 0.2, Platelets 84; peak Ferritin 6283, d-dimer 50.60 and PCT >100, plateauing by day 7.

He was ventilated for 23 days requiring high pressures (PIP consistently >30), nitric oxide and inotropes. There was no clinical improvement despite optimal supportive treatment, but immunomodulation was held during repeat microbiology investigation and concern for losing a fine care balance. However, when formally considering ECMO, a trial of Anakinra 4 mg/kg increasing to 8 mg/kg twice daily over 4 days (day 5–9) was commenced. The inflammatory markers had already plateaued then started to fall and there was no meaningful respiratory improvement with anakinra. Intravenous immunoglobulin (IVIG) was administered day 9–10, but improvement in respiratory function only occurred with IV methylprednisolone 10 mg/kg/dose when (days 16–18) the team felt immunomodulation was safe and data from adult intensive care was supportive. He transferred to the ward on day 30 and discharged to his local hospital on day 83. PCR remained positive until day 32.

**Case report - Discussion:**

As in other children with evidence of SARS-COV2, this child showed a rapid and unpredictable decline in condition, but he presented with an opportunity for prevention of the ensuing hyperinflammation. There is a need for research to identify a fingerprint of inflammation that predicts subsequent decline and the value of prompt, safe immunomodulation to distract from critical cytokine cascades.

It is unclear why this boy was one of very few children to develop an adult phenotype but given an altered immune state associated with trisomy 21 and a previous episode of ARDS with hyperinflammation it would suggest that inherent Trisomy 21 genetic risk factors may be involved. He also had previously been identified to features of chronic lung disease that was a contributing factor.

Successful management of this child resulted from rigorous organ support and close collaboration between consultants and junior staff in PICU, infectious diseases and rheumatology.

This was the beginning of a critical relationship that allowed rapid sharing of information and concerns to maintain safety and explore differentials and treatment strategies. A decision to commence immunomodulation had to consider the fragile state of support and risk; for instance, from fluid overload, the background risk of infection and neoplasia and professionals understanding of drug safety. Anakinra was used first due to a short half-life, extensive safety data in toxic shock and routine use in rheumatology. IVIG was delayed due to fluid volume, but neither this nor anakinra had a meaningful clinical effect. The key response to steroids is like that reported in adults including the RECOVERY trial.

Other learning points showed unexpected patterns and limited utility of blood investigations, as seen later in PIMS-TS. This included incongruence in peaks of ferritin and LDH and delayed recovery of cell lines except Hb which was an early signal of improvement.

**Case report - Key learning points:**

A collaborative approach across teams during the care of this patient was critical and primed relationships prior to the surge of PIMS-TS cases. The role of rheumatology has been clearly defined by its familiarity with the benefit and safety of immunomodulatory agents, which has enabled support of intensive care and infectious disease teams in their use.

This patient may also have highlighted a predisposition in some cohorts to states of hyperinflammation given his previous episode last year. Is this due to his trisomy 21 and suspected immune dysregulation or other factors, as we know these patients are at an increased risk of infections and autoimmune conditions?

Clinical course in children is unpredictable and frequently associated with rapid decline. Teams must remain flexible and able to respond with different treatment strategies as cases continue to evolve. Here there was an adult phenotype and supportive treatment remained key. As in adult care the benefit of steroids in ventilated patients was clear. However, ongoing research is required to examine the safety (and benefit) of early immunomodulation use when other differentials (including sepsis and malignancy) have not been fully or reliably excluded.

There is an argument to use immunomodulation early in the presentation of similar patients in the future. Further molecular biological studies such as the DIAMOND study may help us identify key inflammatory pathways and subsequently screening tools to inform such initial treatment and avoid the rapid decline and hospital or PICU admission. Each immunomodulator has distinct characteristics and response to treatment is not predictable. Does this inform treatment algorithms in the absence of evidence from trials? In the absence of trials for this phenotype should treatment follow an adult based algorithm.

We hope that by sharing this case it will stimulate further discussion and research to help us treat future similar patients.

